# Dosimetric investigation of small fields in radiotherapy measurements using Monte Carlo simulations, CC04 ionization chamber, and razor diode

**DOI:** 10.1007/s13246-025-01546-w

**Published:** 2025-06-16

**Authors:** Mina M. Habib, Mahmoud H. Abdelgawad, Albert Guirguis, Majed Alharbi, Kareem El-Maraghy, Abdelsattar M. Sallam

**Affiliations:** 1 Physics Department, Faculty of Science, Ain Shams University, Cairo, Egypt; 2 Physics Department, Faculty of Science, Al-Azhar University, Cairo, Egypt; 3 Institute for Frontier Materials, Deakin University, Waurn Ponds, Geelong, VIC 3216 Australia; 4 Physics Department, College of Science, Jouf University, Sakaka, KSA Saudi Arabia; 5 Department of Radiation Physics, Maadi Armed Forces Medical Compound, Cairo, Egypt

**Keywords:** Monte Carlo (EGSnrc code), Monaco treatment planning system (TPS), Razor diode, Small fields, Elekta versa HD, Gamma passing rate (GPR)

## Abstract

The motivation of this study is to check the dosimetry of small field sizes used in various treatment techniques using different methods (Monte Carlo simulations and detectors). We created two Monte Carlo models for Elekta Versa HD linear accelerators using EGSnrc (BEAMnrc-DOSXYZnrc) codes. Previous studies led us to define one model with an ideal symmetry full‐width‐half‐maximum (FWHM) of 0.15 cm in the x and y directions for the Gaussian distribution of the primary electron source and redefine the other with a larger asymmetry FWHM of 0.35 cm in the X and 0.6 cm in the Y directions. We calculated the penumbra width using both models. We measured output factors using two different detectors including Razor Diode which is designed especially for small field size measurements and compared them with both models. Using these detectors aims to investigate different detector sensitivities for dose measurements. In addition, patient-specific planning quality assurance (PSQA) for four fictional cases using Elekta Versa HD with Nasopharyngeal, Astrocytoma, right cerebellum, and right breast cancers were done using an IBA—2D array and compared to the minimum segment width parameter in Monaco Treatment planning system (TPS) for (0.5 and 1) cm segment width. The results indicated that Monte Carlo simulation shows increasing in the values of penumbra width with increasing the size of FWHM for field size range 0.5 × 0.5 to 3 × 3 (in-plane: 0.33 to 0.45 for model 1 and 0.46 to 0.65 for model 2, cross-plane: 0.29 to 0.38 for model 1 and 0.44 to 0.62 for model 2). The results indicate that output factors decrease as FWHM increases. The Razor Diode and CC04 detectors show consistent results up until a field size of 1 × 1 cm^2^. Additionally, plans with a minimum segment width of 0.5 cm demonstrate a lower gamma passing rate (GPR) compared to those with a 1 cm segment width. In conclusion, Inaccurate modeling of the FWHM of the primary source can lead to a significant error in the calculation when using a Monte Carlo model of the beam; Accordingly, this may lead to inaccurate delivery of treatment dose for cancer patients, in addition, this error increases as we go down field size 1 × 1 cm^2^ to reach an unacceptable level in field size 0.5 × 0.5 cm^2^. Thus, and as found, we can conclude that: to produce a more accurate radiotherapy treatment plan which in turn will lead to high-quality treatment for cancer patients, It is recommended that, during the beam-shaping process in IMRT or VMAT optimization, the minimum dimensions of any individual beamlet or segment within the treatment field should not be smaller than 1 × 1 cm^2^.

## Introduction

There are some reported technical issues related to the treatment of small field sizes in radiotherapy. One of these issues arises is the loss of lateral charged particle equilibrium along the beam axis. This occurs when the collimating device partially obstructs the primary photon source in the beam path. Additionally, if the size of the detector is the same as or larger than the beam, it can further complicate the measurement process and affect the accuracy of the results [1]. It is even more crucial for stereotactic radiosurgery and Stereotactic body radiation therapy owing to the high dose used in the treatment of the small sized tumor lesions. Any variation in the output will impair the observations of dose response and the optimizations of the prescribed dose [1, 2]. This problem of reading differences between different detectors becomes clear when measuring some of the factors included in the treatment planning software. These factors include but are not limited to measuring the total scatter factors (output factor), which is considered one of the most important factors necessary for calculating dose distribution in treatment planning systems.

In the case of small field-size measurements, the accuracy of this factor depends mainly on the sensitivity of the detector used. In addition, some studies investigated the effect of using inaccurate output factors in Treatment Planning System (TPS) modeling on patient dose distribution. They reported significant differences between treatment plans with different output factor modeling, especially in patient-specific stereotactic treatment plans with small target volumes. Thus, small fields are associated with greater uncertainty in the accuracy of beam [3] modeling and clinical dosimetry [4]. The problem becomes more apparent when more complex treatment plans are applied; for example, intensity-modulated radiotherapy (IMRT) and volumetric arc therapy (VMAT) plans are more complicated to deliver because the design of these plans includes several small subfields (segments) and steep dose gradients in each field of these plans as well. Therefore, the dose distribution obtained from the TPS is necessary to be checked using dosimetric systems to ensure the accuracy of treatment and detect any errors that may occur. Accordingly, inaccurate small-field dosimetry during TPS commissioning is indeed one of the main sources of dose-delivering error [5–7]. Kairn et al. [8] in a similar study, highlighted that the absence of accurate and easily accessible measurement data could result in errors exceeding 10% in treatment plans involving very small or narrow treatment fields [8].

Fogliata et al. [9] in their work on Acurous XB and AAA algorithms showed that the use of small fields in clinical treatment is challenging because, for several reasons from the modeling point of view, the photon source (that has a finite size and is not a point source) might not be fully visible from a point of measurement [9]. In addition, being partially occluded by the collimating system reduces the primary photon fluence, reaching the measurement point proportional to the focal spot size. This fact has a strong impact on field size and radiation output. Also, they add that the spot size parameter models the physical effect of the finite size of a primary source (bremsstrahlung from the target). This modeling is done by applying Gaussian smoothing to the energy flow of primary photons. In particular, the parameter equals the width of the Gaussian distribution either in the X or Y direction at the isocenter.

Chand et al. [10] mentioned that while most commercially available dose calculation algorithms are effective in most clinical situations, none of them can guarantee an accurate estimation of the dose to tissue [10]. They introduced a model of the agility treatment head installed on the Versa HD linear accelerator, which was obtained using Monte Carlo (MC) simulation. This treatment head consists of different components, and each component affects the resulting x-ray beam differently. The geometrical and physical properties of the target and the beam flattening filter play a crucial role in determining the effective energy of the initial beam. For the dose beam profile, the main reason for the difference between the measured dose and the simulated dose using the Monte Carlo algorithm is the defined size of the primary source. Also, Gholampourkashi et al. [4] found that the main reason for the difference in dose profiles between Monaco and measured EGSnrc was the size of the primary source as modeled in the Monaco beam model. They made the FWHM of the primary electron source’s Gaussian distribution bigger from 0.1 cm to 0.2 cm and got a better dose difference % and average distance to agreement (DTA) between Monaco/film and Monaco/EGSnrc depending on their work on previous studies [11–13].

Based on previous studies that we have reviewed, there are still discrepancies between measured and calculated doses in small fields used in radiotherapy, particularly with more complex techniques such as VMAT and IMRT. These discrepancies exceed the limits set by TRS-483, which states that no correction exceeding 5% is acceptable. To provide a solid foundation for identifying these discrepancies, and to the best of our knowledge, no clear explanation for those discrepancies, we believe that our research is a larger step in explaining these discrepancies. Additionally, there has been limited exploration of how these small fields impact the quality of treatment plans. Therefore, we believe our study, with its specific objectives, can help establish the permissible limits for small fields in treatment planning, thereby reducing potential discrepancies between readings from various radiation detectors.

The objective of this study is to analyze the impact of varying the full‐width‐half‐maximum (FWHM) of the Gaussian distribution of primary electron source parameters on the resulting radiotherapy doses from small field sizes. Additionally, we aim to assess how this variation affects the sensitivity of radiation detectors employed in measuring doses for small field sizes. To achieve these objectives, we conducted a series of comparisons between the dose calculations of certain radiotherapy parameters using two different models of FWHMs. The first one will be modeled based on the ideal definition of the size of the electron source, while the second one will be described using a modified definition and asymmetrical of the size of the same electron source. Both models will be implemented using Monte Carlo simulation code models. The Monte Carlo code utilized in this study is EGSnrc (BEAMnrc-DOSXYZnrc) and is freely available for use. Among the comparisons we made, comparing the output factor to both Monte Carlo models, the output factor was measured with different detectors varying in volume. It is important to note that this study is based on a generalized Monte Carlo simulation model of a standard Elekta Versa HD LINAC and is intended for theoretical analysis only. Machine-specific commissioning, including post-modeling adjustment, as recommended by Elekta, was performed separately by the local physics team for clinical implementation but was outside the scope of this study. Therefore, the findings presented here are not directly applicable for clinical treatment planning without local validation and adjustment. Another aim of this work is to detect the effect of the small field size on the quality of treatment plan delivery. This will be done by testing the Gamma Passing Rate (GPR) of some treatment plans and detecting the effect by modifying the small beam segments to enhance the low gamma-passing plan, some previous studies shows that using minimum segment width 1 cm is results in better plan quality Nithiyanantham et al. [14] Showed that increasing the segment width improves plan quality and delivery efficiency for VMAT-SBRT. Jiménez-Puertas et al. [15] Found that minimum segment width = 1.0 cm optimally reduces MU and treatment time without compromising plan quality. They reported that two cases planned with minimum segment width = 0.5 cm failed to meet gamma pass criteria (76.2% and 85.3%), but re-planning with minimum segment width = 1.0 cm resolved these issues.

## Materials and methods

### Specification of the treatment facility and its treatment head

**Fig. 1:**
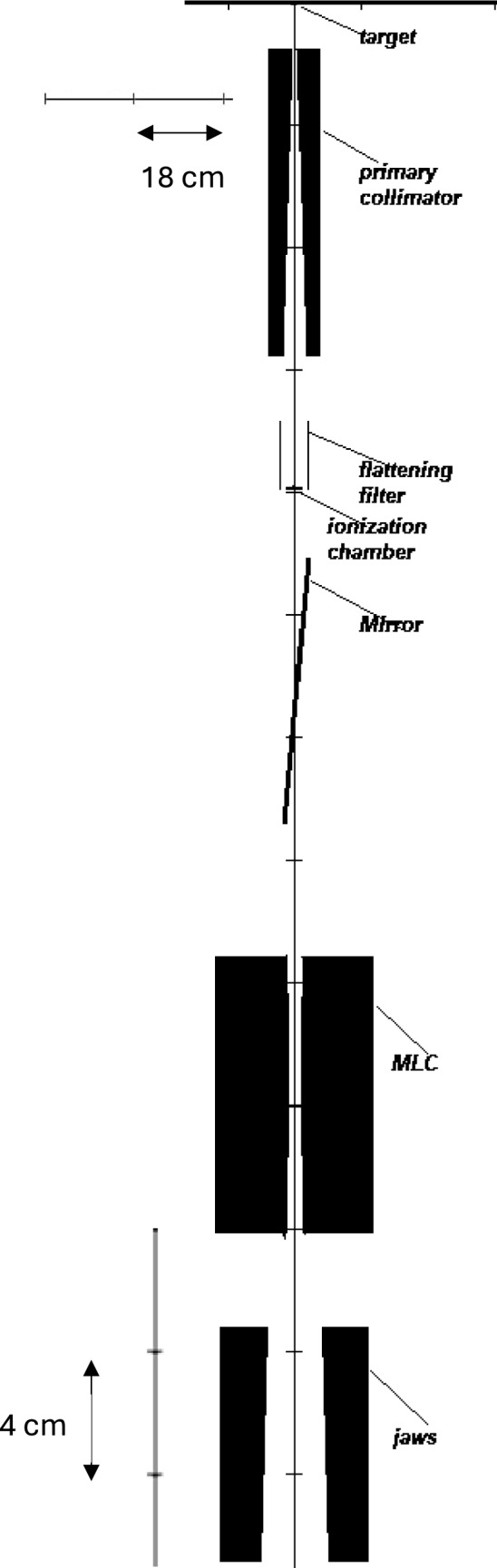
Preview of the component modules used to model the Elekta Versa HD. The preview of the accelerator shown is in the X–Z plane,with Z being the direction of the beam

The Elekta Versa HD linear accelerator (Elekta AB, Sweden), manufactured in 2016, is equipped with an Agility head featuring a Multi-Leaf Collimator (MLC) system. The Agility MLC comprises 160 leaves, evenly distributed between two banks. Each leaf has a width of 0.5 cm, allowing for a maximum field size of 40 × 40 cm^2^ and a minimum field size of 0.5 × 0.5 cm^2^. Unlike other Elekta collimators, such as the MLC2, the Agility system does not include backup jaws. The leaf speed ranges from 3.5 to 6.5 cm/s, with a minimum gap of 3 mm between opposing leaves. Additional parameters include a leaf groove width of 0.4 mm, and a static leaf gap of 0.10 mm as set in the Monaco TPS. The Agility MLC features rounded leaf ends to optimize beam shaping, and the Tongue and Groove effect, a result of the interlocking leaf design, has been minimized to enhance dosimetric precision. The schematic diagram of the Linac head has been demonstrated in Fig. 1.

### Simulation code

In this work, we used EGSnrc (BEAMnrc-DOSXYZnrc) as a free online code with a Monte-Carlo-based simulation algorithm (National Research Council of Canada, Ottawa, ON, Canada) to create two models to find the effect of the FWHM value of the electron beam source along both directions on the specification of delivered treatment beams [15]. The first model was set as the ideal symmetrical model direction with a defined electron source Gaussian distribution equal to 0.15 cm for both X and Y directions based on the obtained previous studies [4, 6, 10]. To check the effect of the electron source diameter value on the results of FWHM, we redefined this parameter in the second model to be large and asymmetrical 0.35 and 0.6 cm in the X and Y directions, respectively [11–13]. For simplification, we called the first model the ideal one, M1, while the second model the modified one, M2.

The selection of the full width at half maximum (FWHM) value for the first model, M1, which we considered ideal, was based on averaging the values provided by various references [4, 9, 10, 12, 16]. These references reported different FWHM values in both directions for various simulated radiotherapy treatment machines. In contrast, the values for the second modified model, M2, were chosen to reflect suggested modifications that exceed the ideal values. As mentioned earlier, Elekta Versa HD linac was used in this study. A 6 MV as a high megavoltage X-ray beam energy was used in the beam delivery and beam simulation. This linac has the option of choosing between beams with flatting filter (FF) and flatting filter-free (FFF) beams for treatment. In this study, we used “6 MV-FF” beam for dose calculation and dose delivery.

The simulated measurements using the two models were the penumbra of the beam, the beam profile, and dose output factors at 10 cm in depth [10]. 2 × 10^8^ histories were simulated in BEAMnrc for generating phase-space files scored at 100 cm. The values of electron cut-off energy (ECUT) and photon cut-off energy (PCUT) have been set at 0.7 and 0.01 MeV, respectively. PRIESTA-II electron spin algorithms have been employed with a maximum step size of 1 × 10^10^ cm. The variance reduction technique has been applied to enhance the accuracy of simulation and reduce simulation time, as the electron range rejection was set to 2 MeV the bremsstrahlung splitting was set to uniform. In the early stages of the simulation, we didn’t use the variance reduction technique, and the electron range rejection was set off, as a result, the simulation ran slower. In addition, the calculated doses via the used code were done based on a water phantom simulation. In DOSXYZnrc 1.1 × 10^10^ histories were applied for each dose calculation to get statistical uncertainty below 0.5%.

To accurately simulate the dose distribution cross section data calculated using EGSnrc simulation,we used (700 ICRU) file where stopping power values recommended by ICRU for different composition were used in the simulation [17].

The simulation calculated by the *Bibliotheca Alexandrina Supercomputer Facility,* Alexandria, Egypt [18].

### Delivering parameters

The energy used in this study was released through the following fields: 3 × 3, 2 × 2, 1 × 1, and 0.5 × 0.5 cm^2^. These fields were used to simulate the practical small field sizes. All examined small fields were defined according to the concept of ${{\varvec{S}}}_{{\varvec{c}}{\varvec{l}}{\varvec{i}}{\varvec{n}}.}$ which is used to define the clinically relevant small field size. This concept is provided in IAEA technical reports, particularly in code No. TRS-483. This report provides recommendations for the dosimetric handling of small fields, particularly in the context of IMRT, VMAT, and stereotactic radiosurgery and/or radiotherapy (SRS & SRT), where small fields are commonly used to deliver high-precision doses to tumors [2].

The common reference field size for Elekta versa HD is 10 × 10 cm^2^ as the reference field size for large field sizes; for small field sizes, it is recommended to select a smaller field size as a reference for the radiotherapy measurements. In this work, we chose 3 × 3 cm^2^ as the reference field size based on prior research. In addition, the field size of 3 × 3 cm^2^ is the smallest field size that can ensure sufficient lateral charged particle equilibrium [19]. This reference size facilitates standardized measurements and consistency in dose calculations, enabling small-field treatments—especially in advanced modalities like Stereotactic Radiosurgery (SRS) or Stereotactic Body Radiotherapy (SBRT) to be delivered with high precision. In addition, we used a 3 × 3 cm^2^ field size as the reference field for output calculations. Also, the simulation and the measurements were done based on a 0° collimator and gantry linac dose delivery.

### Radiation detectors

In this study, we used different detectors, manufactured by IBA Dosimetry, Germany. as detectors used for measurements applied to radiotherapy treatment doses. Table 1 shows the specifications of the detectors used.

**Table 1:** Classification of used detectors

Category	Label	Active volume mm^3^	Materials	Sensitivity
Micro	Razor-Diode	0.6 mm (active detector diameter)	Silicon	4.1 nC/cGy
Mini	CC04	40	Outer and inner Electrode Shonka C554 with tissue-equivalent plastic (1.76 g/cm^3^)	1 nC/cGy

Razor-diode is unshielded detector used for small field sizes, such as 0.5 × 0.5 cm^2^, as provided by the vendor, The Razor detector for relative dosimetry is a very small sized, rigid and long-lasting semiconductor detector with high dosimetric performances. To compare planned to measured doses, we utilized the IBAMatriXX Evolution 2D array. The MatriXX Evolution consists of 1020 air vented pixel ionization chambers uniformly distributed among an area of 24 × 24cm^2^ at 100 cm SDD. The distance between the individual detectors is 7.6 mm (center to center). The MatriXX Evolution includes a temperature and pressure sensor to perform an automated k(t,p) correction of the chamber signal. The parallel readout of all 1020 detectors with a minimum sampling frequency of 20 ms enables acquisition of both, individual IMRT segments as well as the total integrated delivered dose, as provided by the vendor the MatriXX Evolution is a 2D detector array optimized for fast and accurate verification of rotational delivery IMRT beams versus planned data as well as Linac Machine QA, Its software performs interpolation between detectors to enhance resolution for small segments. The device undergoes calibration every quarter using the dose-to-detector calibration method.

### Field output factor

In radiotherapy measurements, the determination of the field output factor—also known as total scatter factors or relative dose factors—is an essential part of the dosimetry calibration process. It is measured by using an appropriate detector, usually placed in a phantom, at a consistent depth and under reproducible conditions. The procedure typically involves irradiating both small and larger fields and calculating the dose ratio. Accurate measurement of the field output factor ensures proper dose delivery, especially in advanced treatments like IMRT, where distribution is highly dependent on field size and shape. According to TRs-483 [2], The field output factor is defined as the ratio of the absorbed dose to water (measured from the chamber reading) within the clinical field ${{\varvec{f}}}_{{\varvec{c}}{\varvec{l}}{\varvec{i}}{\varvec{n}}}$***,*** where the beam quality is denoted as ${\boldsymbol{\varrho }}_{{\varvec{c}}{\varvec{l}}{\varvec{i}}{\varvec{n}}}$, in comparison to that within the reference field ${{\varvec{f}}}_{{\varvec{m}}{\varvec{s}}{\varvec{r}}}$,with beam quality represented as ${\boldsymbol{\varrho }}_{{\varvec{m}}{\varvec{s}}{\varvec{r}}}$. This field output factor ${\boldsymbol{\Omega }}_{{{\boldsymbol{\varrho }}_{{\varvec{c}}{\varvec{l}}{\varvec{i}}{\varvec{n}}}.\boldsymbol{\varrho }}_{{\varvec{m}}{\varvec{s}}{\varvec{r}}}\boldsymbol{ }}^{{{{\varvec{f}}}_{{\varvec{c}}{\varvec{l}}{\varvec{i}}{\varvec{n}}}.{\varvec{f}}}_{{\varvec{m}}{\varvec{s}}{\varvec{r}}}}$ can be computed from the following relationship:

${\boldsymbol{\Omega }}_{{{\boldsymbol{\varrho }}_{{\varvec{c}}{\varvec{l}}{\varvec{i}}{\varvec{n}}}.\boldsymbol{\varrho }}_{{\varvec{m}}{\varvec{s}}{\varvec{r}}}\boldsymbol{ }}^{{{{\varvec{f}}}_{{\varvec{c}}{\varvec{l}}{\varvec{i}}{\varvec{n}}}.{\varvec{f}}}_{{\varvec{m}}{\varvec{s}}{\varvec{r}}}}=\frac{{{\varvec{M}}}_{{\boldsymbol{\varrho }}_{{\varvec{c}}{\varvec{l}}{\varvec{i}}{\varvec{n}}}}^{{{\varvec{f}}}_{{\varvec{c}}{\varvec{l}}{\varvec{i}}{\varvec{n}}}}}{{{\varvec{M}}}_{{\boldsymbol{\varrho }}_{{\varvec{m}}{\varvec{s}}{\varvec{r}}}}^{{{\varvec{f}}}_{{\varvec{m}}{\varvec{s}}{\varvec{r}}}}}{{\varvec{K}}}_{{{\boldsymbol{\varrho }}_{{\varvec{c}}{\varvec{l}}{\varvec{i}}{\varvec{n}}}.\boldsymbol{\varrho }}_{{\varvec{m}}{\varvec{s}}{\varvec{r}}}\boldsymbol{ }}^{{{{\varvec{f}}}_{{\varvec{c}}{\varvec{l}}{\varvec{i}}{\varvec{n}}}.{\varvec{f}}}_{{\varvec{m}}{\varvec{s}}{\varvec{r}}}}$, Where ${{\varvec{K}}}_{{{\boldsymbol{\varrho }}_{{\varvec{c}}{\varvec{l}}{\varvec{i}}{\varvec{n}}}.\boldsymbol{\varrho }}_{{\varvec{m}}{\varvec{s}}{\varvec{r}}}\boldsymbol{ }}^{{{{\varvec{f}}}_{{\varvec{c}}{\varvec{l}}{\varvec{i}}{\varvec{n}}}.{\varvec{f}}}_{{\varvec{m}}{\varvec{s}}{\varvec{r}}}}$ is output correction factor.

This equation shows that the ratio of two readings from different fields does not only give the field output factor. To find the correct field output factor, this ratio must be multiplied by an output correction factor. The TRS-483 tables provide output correction factors for various detectors, but the output correction factor for the Razor diode is not included in this code of practice. This is because the data in TRS-483 comes from published studies over the past decade, some of which may be outdated due to improvements in detector technology.

Additionally, the output correction factor for CC04 is not available at a nominal field size of 0.5 × 0.5 cm^2^ in this code Chi et al. [20] compare the output factors for different field sizes using various datasets of correction factors (TRS-483 and Casar’s published work) and found good agreement for the 6 MV with a wide flattening filter (6MV-WFF) between the two datasets [20]. These findings are also supported by Morales et al. [21]. Consequently, we incorporated additional information from the published works of Casar, Chi et al., and Morales et al. [22, 23] in our calculations.

### Small field size measurement setup

The Blue Phantom^2^ water phantom, provided by IBA, Germany, was utilized for small field size measurements. Initially, the water phantom was positioned under the LINAC’s head to simulate the treatment area. The phantom was then aligned with the LINAC’s central axis to ensure precise beam delivery. The detector, mounted using an appropriate IBA holder, was carefully positioned vertically along the x-axis, ensuring that its active area was perpendicular to the beam. After filling the phantom with water to replicate tissue conditions, the detector was aligned with the cross lines of the treatment field to accurately represent the irradiated volume. The detector was then placed at the desired depth within the phantom, considering the small field size and its geometry, to ensure accurate measurement of the dose distribution. Proper calibration and alignment were confirmed to ensure the accuracy of the measurement process and to minimize any potential positioning errors.

The measurements were carried out in step-by-step mode with optimized acquisition settings: 2–3 s measurement time, 0.5–1 s stabilization, and 0.5–1 cm/sec scan speed. The diode operated in grounded mode without a reference detector, and normalization was done at Dmax or measurement depth depending on the scan type.

### Treatment planning

To investigate the second aim of this work, which is related to the effect of small segments on the quality of beam delivery, we contoured four fictional cases. The cases of Nasopharyngeal, Astrocytoma, right cerebellum, and right breast cancers were selected to represent cases with different planning complexities. Head and neck cases need a high degree of modulation due to the proximity of different organs at risk, which means more smaller segments. On the other hand, the breast case can be an example of a plan with less challenge in contouring and planning.

Advanced treatment techniques, including IMRT and VMAT, were employed for each case.

A suitable treatment technique, either IMRT or VMAT, was selected based on the specific requirements and feasibility of each approach to effectively achieve the treatment objectives. These objectives included delivering the prescribed dose accurately to the target volume while minimizing the impact of scattered radiation on the surrounding healthy tissues. The choice of technique was guided by the need to optimize the dose distribution, ensuring maximum tumor coverage with minimal radiation exposure to adjacent healthy structures.

Specifically, the nasopharyngeal case was treated using a four-arc VMAT technique, while the second case, an astrocytoma, was treated with five equally distributed IMRT fields. The third case, involving the right cerebellum, was treated with six equally distributed IMRT fields. Lastly, the fourth case, a breast cancer case, was treated using a VMAT plan consisting of two partial arcs.

We replanned all cases to magnify the plan modulation to have more small segments in the order of (0.5 to 1) cm. Thus, each case has two advanced plans with similar physical parameters (i.e., number of beams or arcs, angles, and constraints), but they differ in segment width sizes.

We used the Monaco®—treatment planning system manufactured by Elekta, a Swedish company (version 6.1.2.0) with Monte Carlo-based calculation for planning these cases. Monaco treatment planning system developed by (Elekta AB, Sweden) and it is widely used in radiation therapy units. Monaco’s dose optimization algorithm uses both biological models [such as—Tumor Control Probability (TCP) and—Normal Tissue Complication Probability (NTCP)] and dosimetric goals to generate the most effective treatment plans, balancing treatment efficacy with minimizing side effects. It supports dynamic and static segments in IMRT, and for VMAT, it calculates the optimal rotation angles, speeds, and modulations to deliver the best dose distribution. The beam model in the Monaco TPS was pre-configured and validated by the manufacturer, Elekta Sweden. Adjustments to key parameters such as angular divergence, energy spectrum, and spatial distribution were conducted by the physics team at Elekta, ensuring the accuracy and standardization of the Virtual Source Model (VSM). Local reconfiguration of the beam model is not permitted, as this is managed centrally to maintain clinical consistency and compliance with validated protocols.

The selected cases were contoured with a high degree of complexity to declare the effect of parameter small segment width variation from (0.5 to 1) cm. The segment modification in the second plan for each case was done in segments to be not lower than 1 cm in each segment. Each calculated plan (even original or modified) was delivered via versa HD linear accelerator. All delivered plans were received by a 2D array detector to analyze the actual dose distribution. The result of the actual dose distribution of each case was compared with the calculated plan. To compare the two plans, we used the IBA MatriXX 2D array for the isodose distribution comparison. The comparison was done using the gamma passing rate (GPR) formula [6]. GPR calculation is already included in the 2D array software. The agreement criteria were specified as a spatial deviation of 3 mm and a dose deviation of 3%, employing the IBA MatriXX 2D array. According to the established treatment protocol, the Gamma Passing Rate (GPR) threshold is 90%. Consequently, GPR values within the range of 90% to 100% are deemed acceptable, whereas values below 90% are considered unacceptable.

All plans successfully met the acceptance criteria for target volume coverage and organ-at-risk (OAR) dose constraints as per clinical protocols. This ensured that plan quality was maintained while evaluating the effects of segment width variations.

### Patient-specific QA process

Patient-specific quality assurance (PSQA) was performed using the MATRIXX 2D Evaluation dosimetry system and the MULTICube phantom. The phantom, being nearly water-equivalent, was defined in the treatment planning system (TPS) by importing its CT images. IMRT and VMAT QA plans were created with the MatriXX phantom, setting gantry, collimator, and couch angles to zero to ensure static QA as per the study protocol the gantry angle sensor was not used. The QA plan was calculated in the TPS and sent to both the LINAC and my QA patient software -IBA production- for evaluation. In the LINAC room, the phantom was aligned with room lasers, and the MATRIXX detector array was positioned and warmed up using 500 monitor units (MU) to stabilize ionization chambers before measurements. The QA plan was delivered to the MATRIXX system, and the measured dose was compared to the TPS-calculated dose using my QA software, which also provided gamma index analysis (3% DD, 3 mm DTA), dose profiles, and dose distribution comparisons. The gamma passing rate (GPR) normalization in this study was performed using the global method.

## Results

### Effect of modified model on beam profiles

**Fig. 2:**
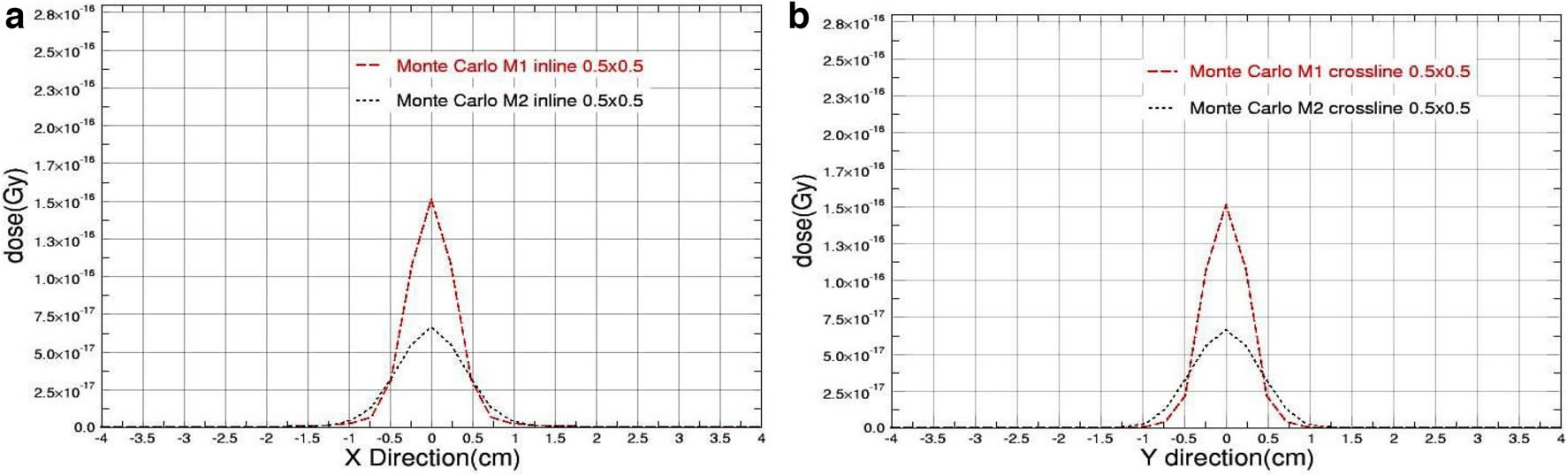
The beam profiles comparison obtained from M1 & M2 Monte Carlo Models for 0.5 cm square field size with **a** in-plane and **b** cross-plane directions

**Fig. 3:**
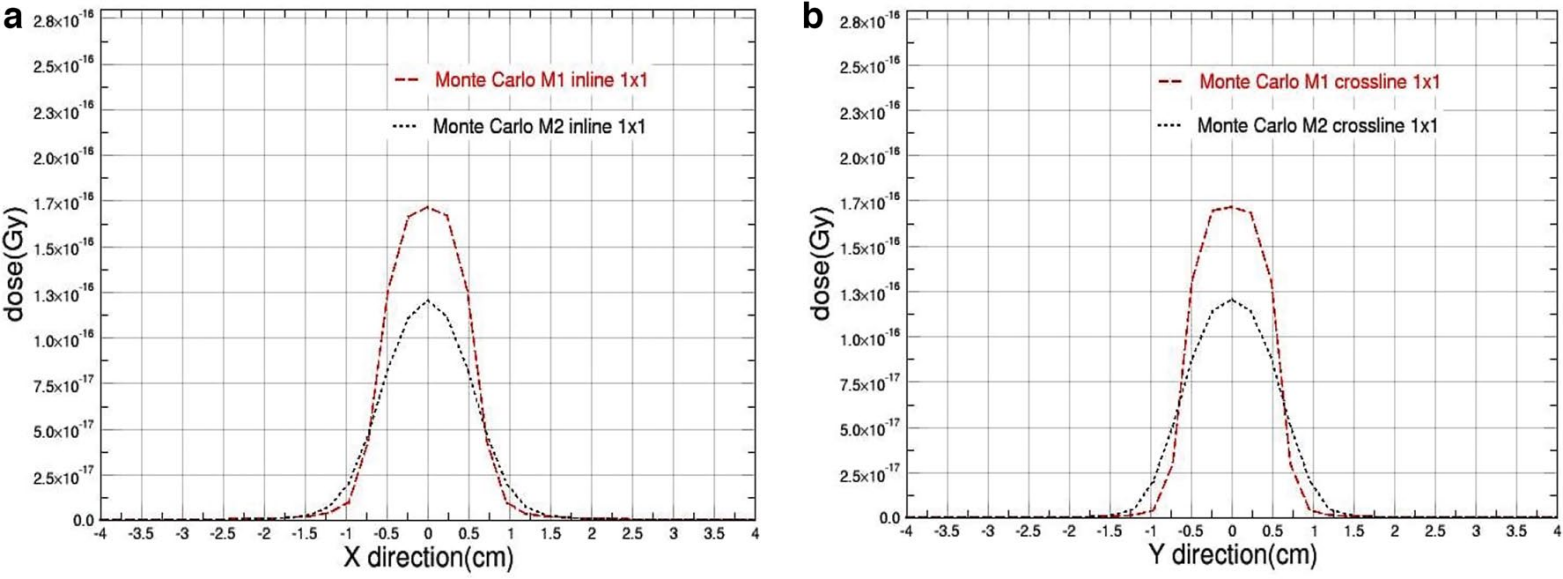
The beam profiles comparison obtained from M1 & M2 Monte Carlo Models for 1 cm square field size with **a** in-plane and **b** cross-plane directions

**Fig. 4:**
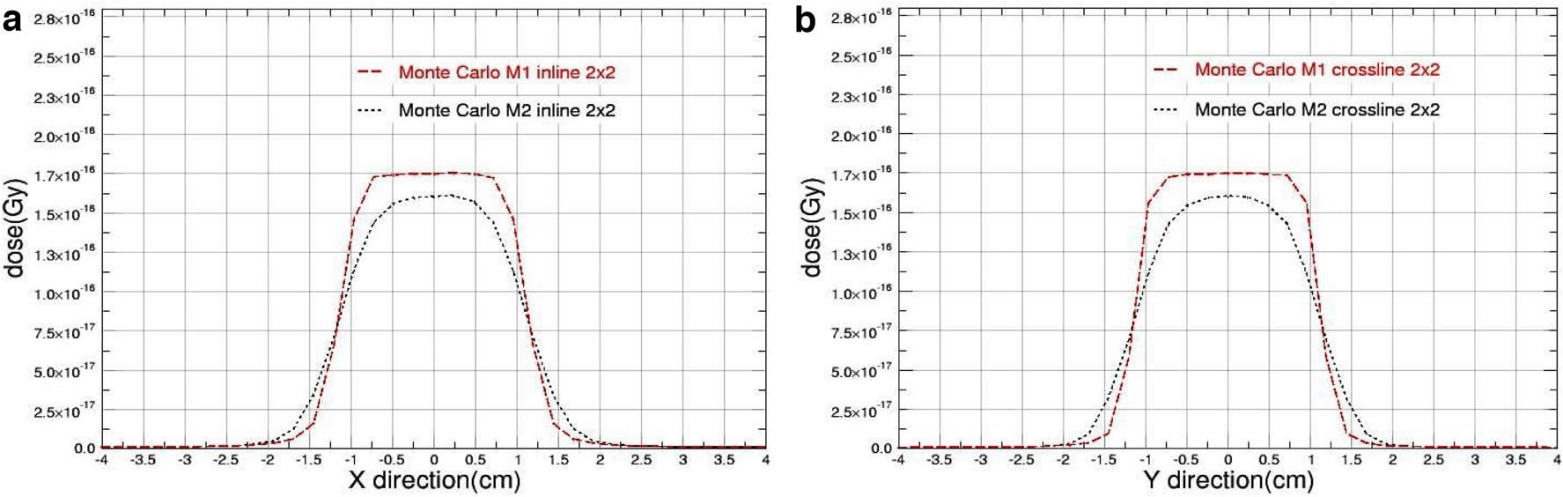
The beam profiles comparison obtained from M1 & M2 Monte Carlo Models for 2 cm square field size with **a** in-plane and **b** cross-plane directions

**Fig. 5:**
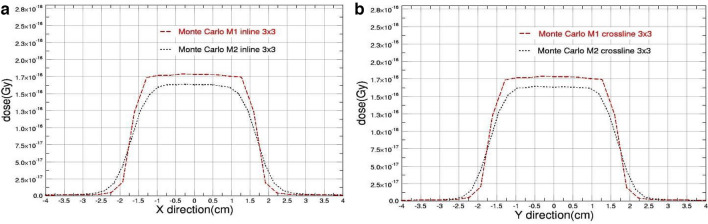
The beam profiles comparison obtained from M1 & M2 Monte Carlo Models for 3 cm square field size with **a** in-plane and **b** cross-plane directions

Figures 2, 3, 4, 5 show the simulation results of the beam profiles obtained from the two defined models. As mentioned earlier, the two models have different definitions in FWHM of the primary electron source; model M1 has a symmetrical size definition in X and Y (0.15 cm) while model M2 has an asymmetrical size definition (0.35 cm in X direction and 0.6 mm in Y direction). The beam profile of 0.5, 1, 2, and 3 square field sizes were indicated in Figs. 2, 3, 4, and 5 respectively. The shown data were plotted using Qtgrace software version 5.1.22.

As shown, the figures clearly demonstrate that the parameter determining the full width at half maximum (FWHM) of the primary electron source significantly affects the outcomes when the field size is decreased.

### Penumbra measurements

The penumbra of field size can be defined as the distance between 20 to 80% of distributed isodose lines [1]. From the previous beam profile curves, we calculated the values of penumbra width for both models for all investigated field sizes. Table 2 indicates the results of simulated penumbra for both models. The percentage difference between both models had been calculated based on the M1 results as the reference result.

**Table 2:** Simulated penumbra with cm of both models (M1 & M2) for 0.5, 1, 2, and 3 square filed sizes

Field size (cm^2^)	In-Plane		Percentage difference (%)	Cross-Plane		Percentage difference (%)
	M1	M2		M1	M2	
0.5 × 0.5	0.33	0.47	35	0.29	0.44	41
1 × 1	0.36	0.54	40	0.24	0.54	76
2 × 2	0.38	0.62	48	0.33	0.60	58
3 × 3	0.42	0.65	43	0.38	0.62	48

As noted, there are significant differences between the results of penumbra obtained from the ideal model (M1) and others obtained from the modified model (M2). On the other hand, the percentage difference between the results of the two models is variable and it is independent of the field size. In general, the average differences between the penumbra results produced from the two models are 41.5 ± 4.7% and 55.7 ± 13% for In-plan and Cross-plane directions respectively.

### Field output factors

**Fig. 6:**
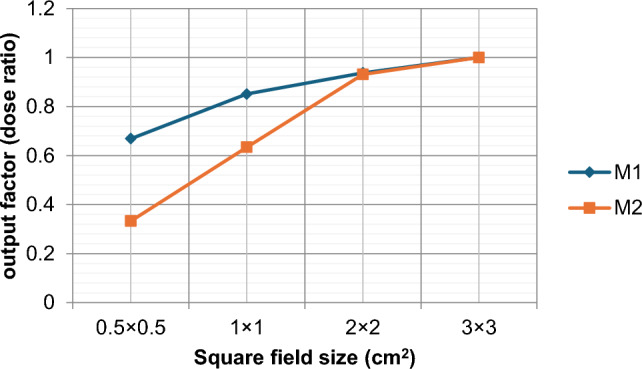
Output factors curves obtained from M1 & M2

Figure 6 shows the output factors curves produced by M1 & M2 models. The simulated output factors were obtained for 0.5, 1, 2, and 3 square field sizes as investigated small fields for 10 cm depth and relative to 3 × 3 square field as reference small field.

As shown in this figure, the output factor increases with increased field size despite the model of simulation. But at the same time, there is a significant difference between the curves in each increase in field size. The maximum diversion between two curves appears in 0.5 square field size with a percentage change equal to a 50% decrease from the ideal model M1.

### Detectors’ measurements

**Fig. 7:**
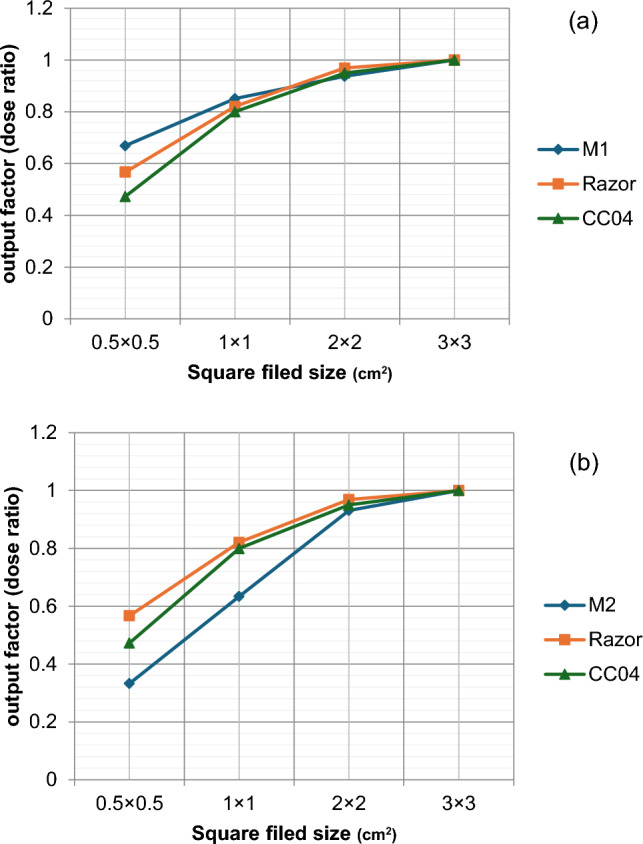
The comparison between output factor measurements using different detectors relative to **a** M1, and **b** M2

In this test, we studied the convergence of both models to actual measurements that can be applied using multiple detectors as a practical application of the validity of both models for therapeutic doses. Figure 7 shows the output factor measurements using Razor and CC04 IBA detectors relative to (a) M1 model and (b) M2 Model.

It is clear from Fig. 7 that the closest results for the output factor calculated for Model M1 are the measurements made using Razor with a percentage change equal to 15% decrease at 0.5 square field size, while the measurements made using the CC04 are considered unrealistic because the difference reached to 30% decrease as a percentage change between them. This is due to the convergence in volume between the ionization chamber to the irradiated field size. Therefore, its results cannot be relied upon for small-field measurements.

The results of the output factor measurements using Razor to output factor calculated for Model M2 had a percentage change equal to a 71% increase at 0.5 square field size. Large variation in output factor registered at 0.5 square field size for the two models and the measurements with different detectors. This result is of great importance to take into consideration, as it is possible to rely on the results of some measurements if they are compared to an incorrect model or if the value of FWHM is modified in it, and consequently, all the results of the treatment planning become subject to correction.

### Patient planning QA

The following table shows the results of GPR agreement between the two plans of each case. As mentioned earlier, the aim of this test is to investigate the effect of small segment width on the quality of Gamma passing rate (Table 3).

**Table 3:** Comparison between GPR of the different minimum segment width parameters

Cases #	Plan #	Minimum segment width (cm)	GPR (%)
Case1	Plane 1	0.5	90
	Plane 2	1	100
Case2	Plane 1	0.5	91.7
	Plane 2	1	100
Case3	Plane 1	0.5	94.5
	Plane 2	1	100
Case4	Plane 1	0.5	87.8
	Plane 2	1	99.7

As shown in this table the value of GPR could be enhanced if the minimum width of segments is not less than 1 cm for criteria (3%–3 mm) for all cases. Our results showed that discrepancies in the measured physical factors will vary among the different dosimetric tools. This poses a challenge in modeling the small fields within the TPS. Inaccurate output factors could lead to incorrect dose calculations. For instance, variations in output factor readings—critical for calculating the appropriate dose—are influenced by the size of the radiation field. Misleading values can lead to substantial risks, resulting in potential underdosing or overdosing of tumors and unnecessary exposure to healthy tissues. Different detectors with varying sensitivity and energy response can contribute to these discrepancies, highlighting the need for rigorous validation of the Treatment Planning System (TPS) and treatment delivery techniques. Additionally, the significance of these findings is evident in Gamma Passing Rate (GPR) results. A high gamma passing rate (≥ 90%) indicates that the measured dose distribution matches the planned distribution, ensuring reliable treatment delivery and minimizing risks to the patient.

In contrast, a low gamma passing rate (< 90%) suggests discrepancies that may reflect errors in treatment setup or dosimetric calibration. Such issues can lead to improper dose delivery and necessitate reassessment of treatment planning and quality assurance protocols, particularly in high-precision treatments like SRS and SBRT, where small errors can have serious clinical implications.

### Uncertainty estimation

The uncertainties in this study were classified into Type A and Type B components. Type A uncertainties, which are quantifiable through repeated measurements or statistical analysis, include the uncertainties from output factor measurements, dose calculations in DOSXYZnrc, and BEAMnrc simulations. Specifically, output factor measurements were repeated five times, yielding a standard deviation ranging from ± 0.003 to ± 0.001, with an average uncertainty of σ_output factor = 0.002. In DOSXYZnrc, 1.1 × 10^10^ particle histories were simulated for each dose calculation, resulting in a dose statistical uncertainty of less than 0.5% (σ_DOSXYZnrc = 0.005). Similarly, in BEAMnrc, 2 × 10⁸ particle histories were simulated, yielding a statistical uncertainty of less than 1% (σ_BEAMnrc = 0.01). The total Type A uncertainty was determined by summing the squares of the individual uncertainties and taking the square root, yielding a total uncertainty of ± 1.14%. Type B uncertainties, which arise from systematic factors such as detector calibration, positioning, and environmental conditions, were minimized through adherence to manufacturer specifications and the application of correction factors based on TRS 483 and previously published data. The typical assumed value for Type B uncertainty was ± 1% (σ_Type B = 0.01). The overall uncertainty for the study was then calculated by combining the Type A and Type B uncertainties using the root sum of squares method. The total estimated uncertainty in this study is approximately ± 1.51% (SD ≈ 0.76%).

## Discussion

The Monte Carlo simulation of particle transport processes is a faithful simulation of physical reality and a powerful tool, as mentioned in previous studies [24]. In radiotherapy, the use of Monte Carlo simulation may be important because it closely approximates the actual results for calculating the therapeutic doses required for patients. However, if the Monte Carlo simulation is based on an incorrect database, this means that the results of therapeutic radiation dose calculations based on this simulation are unrealistic and their values are misleading because they have been compared to incorrect values.

One of the most important data points needed for Monte Carlo simulation in radiotherapy facilities is the full-width-half-maximum value of the electron source. This value may vary from one system to another, and its correct value in simulation has an effect on the convergence of calculation results with measurement results, especially in the case of small fields, which are still under study until now. In order to verify the extent to which the calculation results are affected by the change in the value of full-width-half-maximum (FWHM), this was one of the objectives of this study, in addition to verifying the sensitivity of some radiation detectors to radiation emitted from small fields, specifically those fields with a width of less than 0.5 cm. To verify this, we used two models, as mentioned earlier. Both models differ in the value of FWHM. After running the simulation, we tested the degree of convergence between the actual measurements and each of these two models.

Among these measurements were the measurements of the penumbra area relative to the side length of square field, which showed a large difference between the results in the case of using the modified model. There was an increase in both directions ranging from 55 to 60% in the fields 0.5 × 0.5 cm^2^, while it increased by 28 to 52% in the fields 1 × 1 cm^2^, as well as from 24 to 27% in the fields 2 × 2 cm^2^, and finally from 15 to 16% in the fields 3 × 3 cm^2^. Accordingly, the value of the penumbra increased by increasing the size of the full width at the peak of the main source, and the difference between the two models became larger as the size of the field 1 × 1 cm^2^ decreased, which is consistent with the result of Fogliata et al. [9] Which concluded that the apparent field amplitude was significant for field sizes smaller than 1 × 1 cm^2^ and increased with increasing spot size and also stated that in the case of a fast arc, it was clear even visually that the large smoothing or blurring effect increased with increasing spot size even if acceptable in terms of the calculation of the isocenter output would not be suitable for fast arc. In radiotherapy planning systems for monitoring unit calculations, the dose to the penumbra region is important [25]. This is especially true for radiosurgery which is used by linear accelerators.

Increasing penumbra width and field broadening can result in a decrease in the output factor, as the collimating system partially occludes the primary source. As shown in our results even a slight increase in the primary electron source’s size can cause a significant deviation from the ideal beam model. This deviation escalates when we reduce the field size, to reach an unacceptable level at field size 0.5 × 0.5 cm^2^. We are examining the effectiveness of different detectors in measuring small fields using two different volume detectors, calculating the output factor for field sizes from 3 × 3 cm^2^down to 0.5 × 0.5 cm^2^, and comparing with the output factor obtained from Monte Carlo simulation, large discrepancies founded in output factor values for 0.5 × 0.5 cm^2^ field size for both Monte Carlo model and measurements made with two detectors, increasing field size lead to decrease difference in output factor values, field size 0.5 × 0.5 cm^2^ has a large uncertainty in output factor values. Inaccurate modeling of the full-width-half-maximum of primary electron source can lead a significant error in calculation when using a Monte Carlo model of beam, and this error increase as we go down field size 1 × 1 cm^2^ to reach an unacceptable level in field size 0.5 × 0.5 cm^2^ which agreed with the work of Gholampourkashi et al. [4] variation in detectors reading of output factors in field size 0.5 × 0.5 cm^2^ agreed to TRS-483 [1] that there is no chamber give a true estimation of output factor and even calorimeter need a correction.

Also, some studies explore the difference of dose delivered when using small field sizes, especially 0.5 × 0.5 cm^2^. Ando et al. [26] for instance, evaluated the output factors of two different radiotherapy planning systems using an *Exradin W2* plastic scintillator detector (PSD), and they stated that the Exradin W2-PSD has an output correction factor of 1.0. The TPSs used for dose calculations were RayStation TPS version 10 and Monaco version 5.51.10. A Varian TrueBeam, as the linear accelerator, was used as the treatment machine for calculation and dose delivery. The two TPSs utilized two different calculation algorithms. RayStation utilized the clinical dose calculation algorithm, collapsed cone convolution, while Monaco dose calculation was then performed using the clinical Monte Carlo algorithm, the X-ray voxel. With close agreement with our result, the output factor calculation by Monaco was evaluated using the Exradin W2 (PSD), demonstrating that the practical small irradiation field when the OPF calculation results are within a tolerance of 2.0% is ≧ 1.5 × 1.5 cm^2^, and for field size < 1.5 × 1.5 cm^2^, the pass rate (%) with Monaco is large to reach (23%) for field size 0.5 × 0.5 cm^2^.

Goodall et al. [27], in another study, performed calculations using Monaco TPS. They focus in their work on examining the effect of calculating dose with VOI mean dose in small fields, declaring that it is particularly challenging for small field calculations due to the limited number of voxels within the treatment field and the potentially significant dose gradients across the in-field profile.

Also Lechner et al. [28], aim to test the implementation of small field dosimetry following TRS-483 and to develop quality assurance procedures for the experimental determination of small field output factors (SFOFs), and they recommended that deviations of measured SFOFs to the linac-type-curves of more than 7%, 3%, and 2% for field sizes 0.5 × 0.5 cm^2^, 1 × 1 cm^2^, and field sizes larger than 1 × 1 cm^2^, respectively, should be followed up.

In the final stage of our study and to complete our point of view we use Monaco TPS to create four fictional cases with a different degree of challenge in contouring and planning. For each case, we create two plans with identical parameters except one parameter provided in Monaco TPS called the minimum segment width which this parameter represents the smallest area (cm^2^) you are willing to accept in plane, and as the structure became complex Monaco use small segments to achieve the constraint, in our study using MatriXX evaluation 2D array we calculate the GPR for all plans and found that higher GPR associated with using small segment width (1 cm) than (0.5 cm), lower GPR in segment width (0.5 cm) may related to the discrepancies in output values and penumbra width for field size 0.5 × 0.5 cm^2^.

The Monaco treatment planning system performs a two-phase optimization: Phase I uses a finite size pencil beam algorithm to optimize beamlet weights, followed by Phase II, where segment weights are optimized using the Monte Carlo algorithm. Once fluence profiles are generated, the Static Sequencer processes them through several operations to convert the profiles into deliverable MLC segments. One of the key early steps is the Fill and Skim operation. This step fills narrow fluence valleys (dips) and removes narrow peaks that are smaller than the user-defined minimum segment width. Following this, the Decomposition process acts on the remaining peaks—those not removed during Fill and Skim—and splits them into separately deliverable MLC segments. These segments are generally more regular in shape and are optimized to minimize unnecessary MLC leaf motion. Although these generated segments may appear geometrically small, they differ from the standardized, uniform small fields used in SRS. In VMAT, small segments are irregular in shape, often varying between control points and arc positions. Additionally, their dose contribution is often part of a larger modulation strategy rather than a standalone small field delivery. However, if the optimizer creates a segment with a clear geometric size of 0.5 × 0.5 cm^2^, and it is delivered under static conditions or as part of a narrow segment in VMAT, it will act dosimetrically as a true small field. In this context, the same dosimetric challenges associated with small fields—such as lateral charged particle disequilibrium and detector volume averaging [29]—become relevant, reinforcing the need for accurate modeling and QA in highly modulated VMAT plans. And also, As plan complexity increases and the optimizer struggles to meet clinical constraints, it often introduces smaller segments—even for larger targets. In our breast case, for example, although the target was large, its proximity to organs at risk required high modulation.

## Conclusion

Inaccurate modeling of the full-width-half-maximum of the primary source can lead to a significant error in the calculation when using a Monte Carlo model of the beam and this error increases as we go down field size from 1 × 1 cm^2^ to reach an unacceptable level in field size 0.5 × 0.5 cm^2^ Treatment with field sizes smaller than 1 × 1 cm^2^ used in various techniques such as IMRT and VMAT is not recommended unless the machine and detector commissioning processes are highly reliable. Monte Carlo codes, including EGSnrc, serve as powerful tools that can be effectively employed in the commissioning process and calibration tests. By comparing measured data from linear accelerators and beam models in the TPS, any discrepancies between these models can reveal potential sources of errors. Conclusively, with the continuous advancement of oncology technology and the enhancement of devices capable of delivering precise and targeted tumor treatment, there is a compelling need to prioritize research efforts toward the development of radiation detectors. These detectors should be proficient in detecting high-dose radiation emitted from small fields simultaneously. In the interim, we advocate for further investigation into alternative detection methods, such as diodes, and a thorough assessment of their compatibility with treatment planning algorithms. Moreover, we encourage experts in the field to establish comprehensive guidelines for innovators involved in radiation therapy algorithms, with particular emphasis on the significance of the Full Width at Half Maximum (FWHM) value for the Monte Carlo based systems. This focus is vital for minimizing discrepancies between the radiation measurements captured by detectors and the predictions made by contemporary algorithms.

## Data Availability

Data sets generated during the current study are available from the corresponding author on reasonable request.
